# Critical role of c-Jun overexpression in liver metastasis of human breast cancer xenograft model

**DOI:** 10.1186/1471-2407-7-145

**Published:** 2007-08-01

**Authors:** Yan Zhang, Xiaoyun Pu, Ming Shi, Liyong Chen, Yuhua Song, Lu Qian, Guogang Yuan, Hao Zhang, Ming Yu, Meiru Hu, Beifen Shen, Ning Guo

**Affiliations:** 1 Institute of Basic Medical Sciences, Beijing 100850, PR China; 2 Xinqiao Hospital, The Third Military Medical University, Chongqing 400038, PR China; 3 307 Hospital, No. 8 East Street, Fengtai District, Beijing 100071, PR China; 4 The Department of Oncology, Affiliated Hospital of Qingdao University, Qingdao, Shandong 266003, PR China

## Abstract

**Background:**

c-Jun/AP-1 has been linked to invasive properties of aggressive breast cancer. Recently, it has been reported that overexpression of c-Jun in breast cancer cell line MCF-7 resulted in increased AP-1 activity, motility and invasiveness of the cells *in vitro *and tumor formation in nude mice. However, the role of c-Jun in metastasis of human breast cancer *in vivo *is currently unknown.

**Methods:**

To further investigate the direct involvement of c-Jun in tumorigenesis and metastasis, in the present study, the effects of c-Jun overexpression were studied in both *in vitro *and in nude mice.

**Results:**

Ectopic overexpression of c-Jun promoted the growth of MCF-7 cells and resulted in a significant increase in the percentage of cells in S phase and increased motility and invasiveness. Introduction of c-Jun gene alone into weakly invasive MCF-7 cells resulted in the transfected cells capable of metastasizing to the nude mouse liver following tail vein injection.

**Conclusion:**

The present study confirms that overexpression of c-Jun contributes to a more invasive phenotype in MCF-7 cells. It indicates an interesting relationship between c-Jun expression and increased property of adhesion, migration and *in vivo *liver metastasis of MCF-7/c-Jun cells. The results provide further evidence that c-Jun is involved in the metastasis of breast cancer. The finding also opens an opportunity for development of anti-c-Jun strategies in breast cancer therapy.

## Background

The composite transcription factor activating protein-1 (AP-1) is thought to participate in fundamental cellular processes and control cellular responses to stimuli that regulate proliferation, differentiation, oncogenic transformation and apoptosis [1-5]. AP-1 proteins are composed principally of homodimers of Jun family members (c-Jun, JunB, JunD) or heterodimers of the Jun family members with the Fos family members (c-Fos, FosB, Fra-1, and Fra-2) [6-9]. These homodimers or heterodimers bind to specific DNA sequences, known as 12-Otetradecanoylphorbol-13-acetate response elements (TRE), in the promoter regions of target genes and activate transcription [10-13]. Among them, c-Jun is the major component of the AP-1 complex and c-Fos is its best-known partner. *In vitro *studies have demonstrated that Fos/Jun heterodimers are more stable and efficient in driving transcriptional activation than a Jun/Jun homodimer [14].

c-Jun is frequently overexpressed in human cancers. Increased c-Jun transcriptional activity and AP-1 mediated gene expression are correlated with ras-transformation [15-18]. Since tumor formation and TPA-induced invasion of malignant epidermal cell lines could be blocked in mice expressing a dominant negative transactivation mutant of c-Jun (TAM67), the role of c-Jun in carcinogenesis has been suggested [19,20]. In liver tumors, c-Jun antagonizes p53 by protecting tumor liver cells from apoptosis. It was further demonstrated that c-Jun deletion led to the accumulation of p53, the cyclin-dependent kinase inhibitor p21 and retinoblastoma (Rb), and apoptosis in experimentally induced c-Jun-/- liver tumors [21,22]. The role of c-Jun on angiogenesis in rodents was also described [23].

c-Jun/AP-1 has been linked to invasive properties of aggressive breast cancer [24-27]. Recently, it was reported that overexpression of c-Jun in breast cancer cell line MCF-7 resulted in increased AP-1 activity, motility and invasiveness of the cells *in vitro *and tumor formation in nude mice [25]. The cells expressing dominant-negative c-Jun fail to invade in response to EGF [28]. However, the association of c-Jun overexpression with tumor metastasis *in vivo *has not been demonstrated.

The process of invasion and metastasis depends on the co-ordinate expression and function of a number of gene products conferring the ability of tumor cells to successfully complete a series of events that include breaking away from the primary tumor, invading through basement membrane barriers and the extracellular matrix (ECM) to colonize distant sites in the organism [29]. In the present study, involvement of c-Jun in invasiveness and metastasis both *in vitro *and *in vivo *was investigated by ectopic overexpression of c-Jun in breast cancer cell line MCF-7 and MCF-7/c-Jun xenograft model.

## Methods

### Breast cancer cell lines

Human breast cancer cell line MCF-7 was obtained from American Type Tissue Culture Collection (Rockville, MD) and grown in Dulbecco's modified Eagle's medium (DMEM) supplemented with 10% fetal bovine serum (FCS), 1 mM glutamine and antibiotics.

### Construct and transfection

The cDNA encoding c-Jun protein was amplified by reverse transcription PCR (RT-PCR) with primers 5'-ggaattccaccatgactgcaaagatggaaacgacct-3' (*EcoR*I site underlined) and 5'-cgggatcccgttatcaaaatgtttgcaactgctgcgtt-3' (*Bam*H site underlined) and LA Taq DNA polymerase (TaKaRa). The RT-PCR products were then inserted into the vector pCDNA3.1 by *EcoR*I and *Bam*HI sites (pCDNA3.1/c-Jun). MCF-7 cells were transfected with pCDNA3.1/c-Jun by using Lipofectamin PLUS reagent (Gibco BRL) according to the manufacturer's protocol. The empty vector was used as a control. To obtain transfectants stably expressing c-Jun, the transfected MCF-7 cells were selected in the presence of G418 at a concentration of 800 μg/ml (Sigma). Single neomycin-resistant clones were picked and cultivated in the presence of G418 (200 μg/ml).

### Cell lysate preparation and Western blot

The cells were lysed in ice-cold lysis buffer [50 mM Tris.Cl, pH 7.4, 100 mM NaCl, 10% glycerol, 1% Nonidet P-40, 1 × cocktail protein inhibitors (Roche)] and centrifuged at 10 000 × g at 4°C for 10 minutes. Supernatants were transferred to new tubes. Samples were denatured by being heated to 100°C prior to fractionation onto SDS-PAGE gels. After transfer to nitrocellulose membranes, filters were blocked for 1 hour in blocking buffer [50 mM Tris.Cl, pH 7.5, 100 mM NaCl (Tris-buffered saline, TBS) containing 5% dry milk and 0.2% Tween-20] and then incubated for 1 hour with the primary antibody against c-Jun [c-Jun (H-79): sc-1694, Santa Cruz Biotechnology, Inc.] diluted in blocking buffer. After being washed with TBS (50 mM Tris.Cl, pH 7.5, 100 mM NaCl, 0.2% Tween-20), filters were incubated with horseradish peroxidase-conjugated secondary antibody (Beijing Zhongshan Golden Bridge Biotechnology Co. LTD) for 30 minutes and bands detected by SuperSignal^® ^West Femto Maximum Sensitivity Substrate (PIERCE).

### Proliferation

*In vitro *proliferation of MCF-7 cells was measured over 5 days with an initial cell density of 3.5 × 10^3^/well and six replicate wells/time point by MTT assay as follows. The medium in each well was replaced with 100 μl of medium containing MTT at 0.5 μg/μl and plates were returned to the incubator for 4 hours. The medium-MTT was then removed and 100 μl of dimethyl sulfoxide added to each well. The plates were kept in agitation for 10 minutes in the dark to dissolve the MTT-formazan crystals. The absorbance of the samples was recorded at 570 nm. The results are presented as the mean ± the standard deviation (SD). The experiment was repeated for three times.

MCF-7 cells were washed twice with cold phosphate-buffered saline (PBS) and fixed in cold 70% (v/v) ethanol at 4°C for at least 18 h. Then the cells were harvested and sequentially incubated with RNase A (100 μg/ml) at 37°C for 30 minutes and stained with propidium iodide (PI) (50 μg/ml) in the dark at 4°C for 20 minutes. DNA content was measured by a FACScan cytometer (Becton Dickinson). The experiment was repeated twice.

### Adhesion assays

For adhesion assay, briefly, triplicate wells were precoated overnight at 4°C with BSA (1% w/v), Matrigel diluted 1:8 in DMEM and fibronectin (10 μg/ml). The cells were seeded at 1 × 10^4^/100 μl in serum-free DMEM supplemented with 0.1% BSA. The cells were allowed to adhere for 1 hour at 37°C. Nonadherent cells were removed by gentle washing with PBS and adherent cells were evaluated with MTT method and OD570 nm determination. The experiment was conducted in triplicate.

### Wound migration assay

Cells were cultured in DMEM supplemented with 10% FCS on Matrigel (diluted 1:10 in DMEM)-coated 96-well plates for 24 hours. The injury lines were made with a tip on the cells grown at >90% confluency. After being rinsed with serum free DMEM, cells were allowed to migrate in DMEM supplemented with 1% FCS. The area of cell-free wound was photographed with an inverted microscope (Nikon, Tokyo, Japan) equipped with a digital camera (Spot, Diagnostic Instruments, Inc., Sterling Heights, MI) at indicated time points. The wound healing effect was calculated as the ratio of leading edge to initial wound length. The experiment was performed by duplicate.

### *In vivo *studies

Five-week-old female Balb/C athymic nude mice were purchased from the Institute of Laboratory Animal Science, Chinese Academy of Medical Science and housed in specific pathogen-free conditions in Good Laboratory Practice facility. For all studies, the mice were allowed to acclimate at least 3 days after receipt of shipment and caged in groups of 6. Before injection, the cells were washed with PBS, harvested by trypsinization, resuspended and kept on ice until injection. Precise cell counts were obtained from samples of the cell suspensions by using a hemocytometer. 2 × 10^7 ^MCF-7 or MCF-7/c-Jun cells suspended in 200 μl of PBS were injected s.c. in the right flank. For experimental lung or liver metastasis assays, mice were injected intravenously with 3 × 10^6 ^tumor cells in a volume of 200 μl via the lateral tail vein. Primary tumors, lungs and livers were autopsied 3–4 weeks post injection and tissues with metastases were either photographed for gross morphology or processed for histology as described in the following.

### Histology

Tissues were dissected and fixed in 10% buffered formalin and embedded in paraffin wax. Tissue sections (4 μm) were stained with H & E for morphology. The photographs were taken on a Nikon microscope.

### Statistical analysis

Data were expressed as mean ± SD. For proliferation and adhesion assay, the data were compared by the Mann-Whitney test. For wound migration assay, the comparisons were made by repeated-measures ANOVA as appropriate. *P *< 0.05 was considered statistically significant.

## Results

### Effect of c-Jun expression on the proliferation and cell cycle of MCF-7 cells

**Figure 1:**
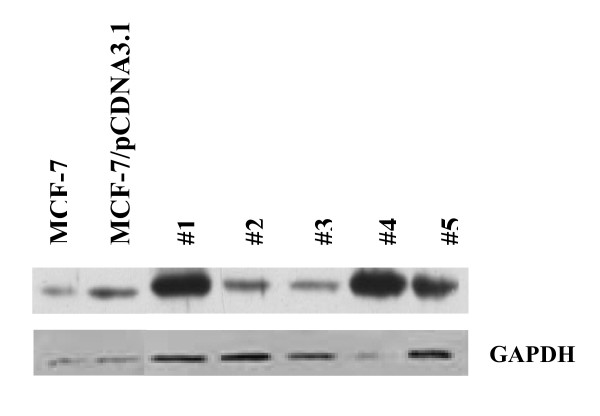
**Western blot analysis of c-Jun expression in individual clones of stable transfectants**. Cell lysates were subjected to SDS-PAGE, transferred to nitrocellulose membrane, and incubated with the primary antibody against c-Jun and horseradish peroxidase-conjugated secondary antibody. Bands were detected by SuperSignal^® ^West Femto Maximum Sensitivity Substrate.

In order to investigate the effects of c-Jun on tumorigenesis and metastasis of breast cancer cells, pCDNA3.1/c-Jun was transfected into MCF-7 cells. The transfected MCF-7 cells were selected in levels of Geneticin (G418) up to 800 μg/ml for stable c-Jun expression. Individual neomycin-resistant colonies (MCF-7/c-Jun) from pCDNA3.1/c-Jun transfections were isolated, expanded and analyzed for c-Jun expression. Western blot analysis of expression of c-Jun protein in individual clones of stable transfectants is illustrated in Fig. 1. Compared to mock transfection, a considerable increase of c-Jun expression with an apparent molecular weight 39 kDa was observed in MCF-7/c-Jun cells. A significant difference in the increase of c-Jun protein expression was found in clones 1 and 4 of stable MCF-7/c-Jun transfectants. Clone 1 was then used in the following experiments.

**Figure 2:**
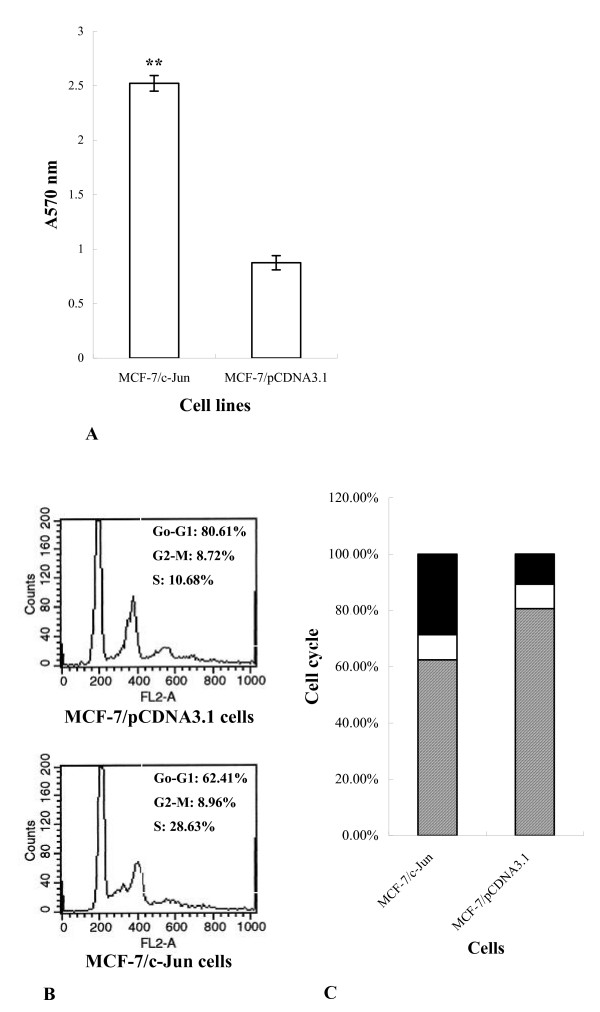
**Effect of c-Jun expression on the proliferation and cell cycle of MCF-7 cells**. A, proliferation assay. Cells (3.5 × 10^3^/well) were cultured for continuous five days and proliferation of MCF-7/c-Jun cells was measured in an MTT-based assay. ** *P *< 0.01 B and C, cell cycle analysis. MCF-7 cells were washed with cold PBS and fixed in cold 70% (v/v) ethanol at 4°C for at least 18 hours. Then the cells were incubated with RNase A (100 μg/ml) at 37°C for 30 minutes and stained with propidium iodide (PI) (50 μg/ml) in the dark at 4°C for 20 minutes. DNA content was measured by flow cytometry. Striped column, G0-G1 phase; empty column, G2-M phase; solid column, S phase.

To evaluate the effect of c-Jun overexpression on the growth of MCF-7 cells, clone 1 was cultured for continuous five days and proliferation of MCF-7/c-Jun cells was measured in an MTT-based assay. Fig. 2A shows that the expression of c-Jun significantly promoted MCF-7 cell proliferation (*P *< 0.01) compared with mock transfected MCF-7 cells. To determine the effect of c-Jun expression on cell cycle progression, the cells were analyzed by PI staining and flow cytometry. A significant increase in S phase cells (from 10.68% to 28.63%) could be observed in MCF-7 cells overexpressing c-Jun, indicating the involvement of c-Jun in positive regulation of cell cycle (Fig. 2B and 2C).

### Effect of c-Jun expression on the adhesion and motility of MCF-7 cells

**Figure 3:**
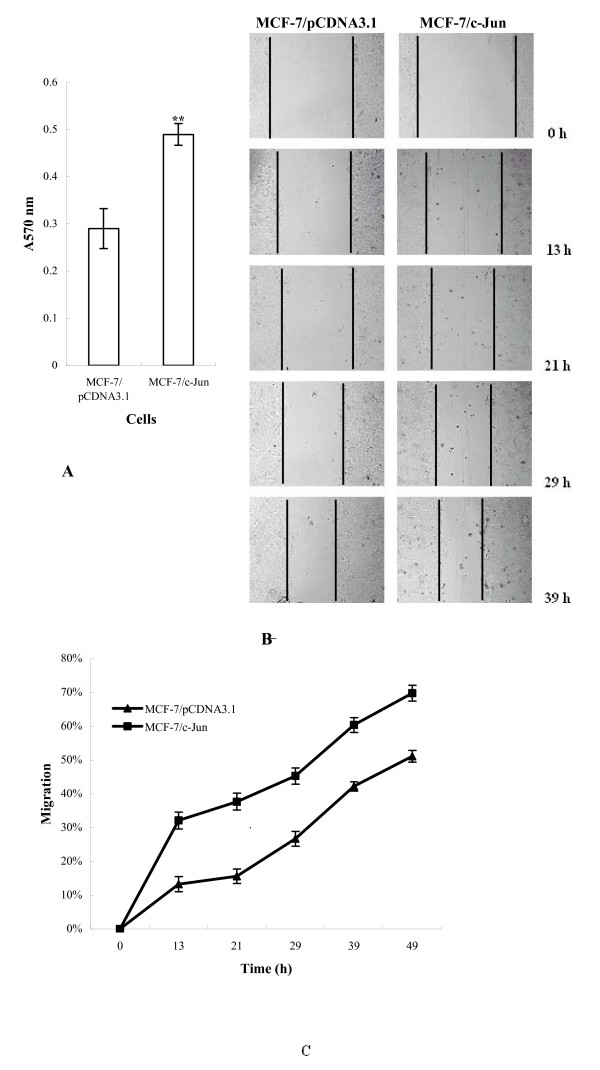
**Effect of c-Jun expression on the adhesion and motility of MCF-7 cells**. A, Adhesion assay. Triplicate wells were precoated overnight at 4°C with BSA (1% w/v), Matrigel diluted 1:8 in DMEM and fibronectin (10 μg/ml). The cells were seeded at 1 × 10^4^/100 μl in serum-free DMEM supplemented with 0.1% BSA and allowed to adhere for 1 hour at 37°C. Nonadherent cells were removed by gentle washing with PBS and adherent cells were evaluated with MTT method and OD570 nm determination. B and C, Wound migration assay. **P < 0.05 Cells were cultured in DMEM supplemented with 10% FCS on Matrigel (diluted 1:10 in DMEM)-coated 96-well plates for 24 hours. The injury line was made on the cells grown at >90% confluency. After being rinsed with serum free DMEM, cells were allowed to migrate in DMEM supplemented with 1% FCS, and photographs were taken at indicated time points. *P *< 0.01

The process of tumor cell invasion involved in tumor cell adhesion to the basement membrane, degradation/proteolysis of the basement membrane and tumor cell migration into secondary sites. Cell movement is generally a continuous and dynamic interplay of adhesions at the leading edge and deadhesions at the rear portion of the cells. A certain degree of cell attachment to ECM substrates is necessary for cell motility. Tumor cell lines that are highly invasive and metastatic exhibit a higher degree of adhesion and motility than their lower metastatic counterparts. To investigate the effect of c-Jun expression on the adhesion and motility of MCF-7 cells, the adhesive ability of MCF-7/c-Jun cells was compared with MCF-7/pCDNA3.1 cells. As shown in Fig. 3A, expression of c-Jun effectively increased adhesion of transfected MCF-7 cells to dishes coated with the Matrigel (*P *< 0.05).

Next the cell motility and the invasive capacity of MCF-7/c-Jun cells were analyzed in a classical wound migration assay. Cell motility into the Matrigel-coated area was determined from micrographs taken at various time points. Compared with the control cells, the motility MCF-7/c-Jun cells was significantly increased (Fig. 3B and 3C), indicating that ectopic expression of c-Jun promoted cell movement (*p *< 0.01).

### Effect of c-Jun overexpression on potential of MCF-7 tumor formation and liver metastases in nude mice

**Figure 4:**
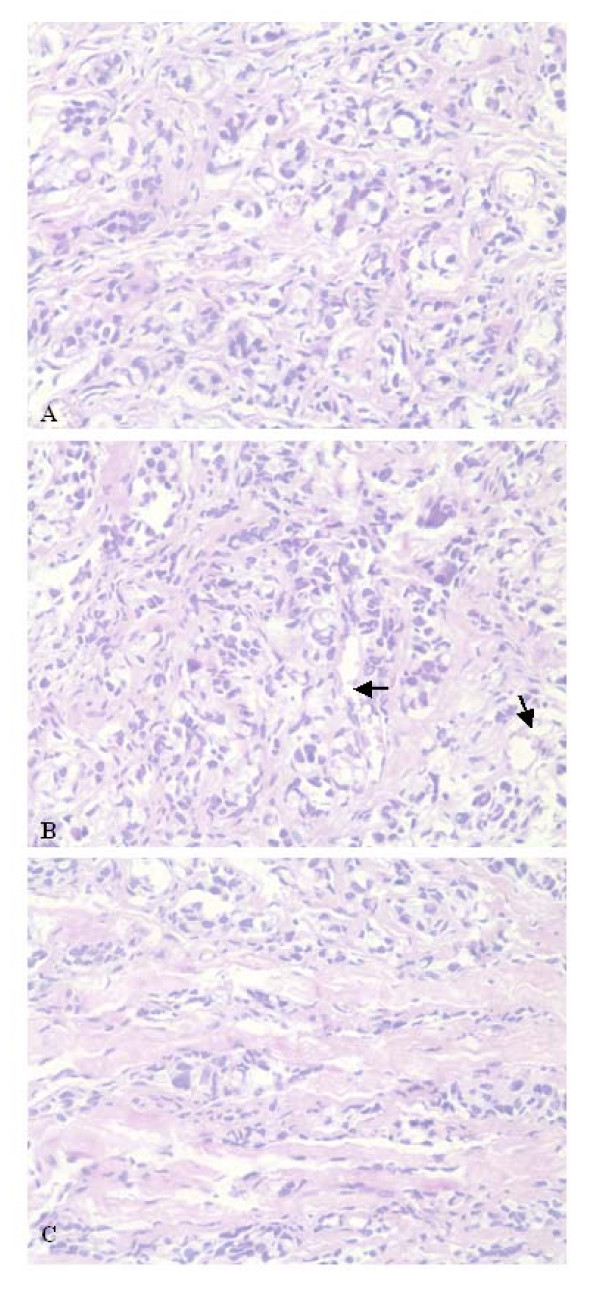
**Effect of c-Jun overexpression on potential of MCF-7 tumor formation**. 2 × 10^7 ^MCF-7 or MCF-7/c-Jun cells suspended in 200 μl of PBS were injected s.c. in the right flank. After 3–4 weeks, tumor tissues were dissected and fixed in 10% buffered formalin and embedded in paraffin wax. Tissue sections (4 μm) were stained with H & E for morphology. The photographs were taken on a Nikon microscope. A, the tumors are composed of relatively large cells that differ in size and display areas with glandula-like arrangement of tumor cells. B, the blood vessels were visualized in the tumor tissues. C, the tumors were highly invasive in the surrounding muscle tissue. ← blood vessels ×400 magnification.

The effect of c-Jun overexpression on tumor formation in nude mice was investigated by injecting 2 × 10^7 ^cells subcutaneously into mice. The tumor growth was observed in 4/6 mice injected with MCF-7/c-Jun cells, but parental MCF-7 cells did not produce tumors. Histologically, the tumors were composed of relatively large cells that differ in size and displayed areas with glandula-like arrangement of tumor cells (Fig. 4A). The overall tumor morphology was essentially similar to that observed in poorly differentiated adenocarcinoma. The tumor cells were separated by bundles of extracellular matrix and blood vessels visualized. The tumors were highly invasive in the surrounding muscle tissue (Fig. 4B and 4C). Mitosis could be found.

**Figure 5:**
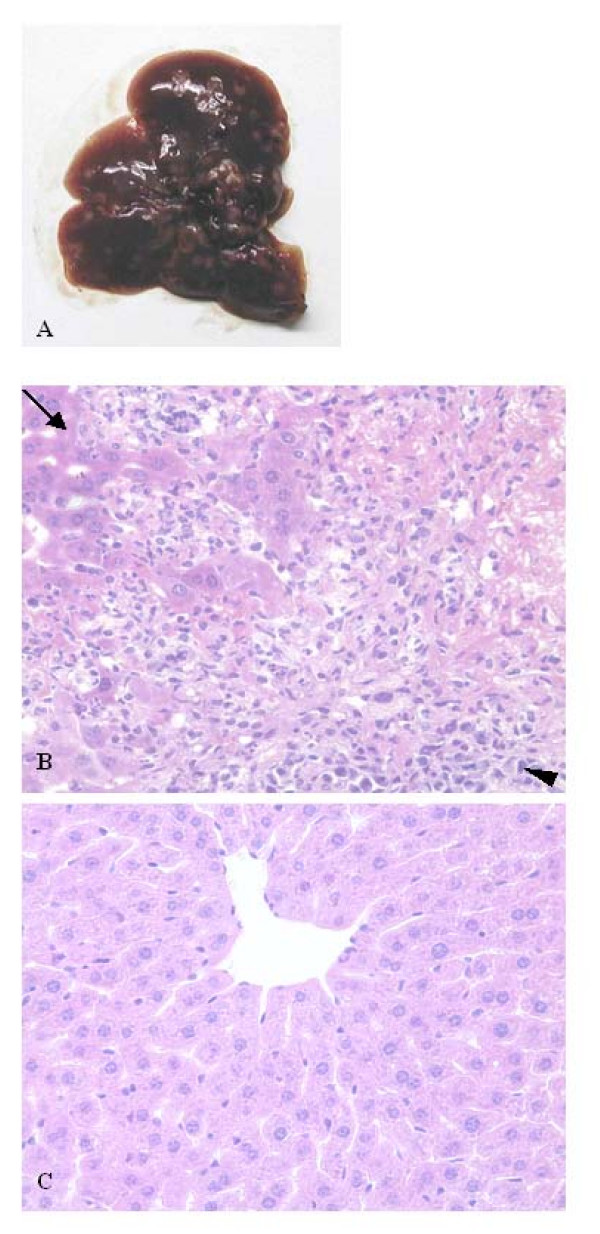
**Effect of c-Jun overexpression on potential of MCF-7 tumor liver metastases in nude mice**. Mice were injected intravenously with 3 × 10^6 ^tumor cells in a volume of 200 μl via the lateral tail vein. The livers were autopsied 3–4 weeks post injection and tissues with metastases were photographed for gross morphology or processed for histology. A, the external image of multiple metastatic lesions of a liver. B, massive infiltration of the liver by MCF-7/c-Jun derived tumor cells observed by microscopic examination. C, no liver infiltration by parental cells was observed. ← liver tissue; ◀ metastasis ×400 magnification.

Then the effect of c-Jun overexpression on the metastatic potential was investigated by injecting MCF-7/c-Jun cells intravenously into nude mice. Six mice were monitored for lung and liver metastases. Metastatic colonization was evaluated by gross examination and microscopic inspection of tissue sections. Three of six mice developed visible liver metastases within 30 days. Livers with MCF-7/c-Jun cell derived metastases were enlarged. A clear external image of multiple metastatic lesions of livers was observed in the mice injected with MCF-7/c-Jun cells by gross examination, but no visible metastatic nodules in the livers of mice injected with MCF-7 cells (Fig. 5A). The livers from each group were removed and processed for histological examination. Microscopic examination of liver tissue sections revealed a massive infiltration of the liver by MCF-7/c-Jun derived tumor cells (Fig. 5B), but no liver infiltration by parental cells was observed (Fig. 5C). The tumor morphology was comparable to that observed in sections from subcutaneous tumors. Tumor cells in metastatic foci included a heterogeneous population of cells consisting of tumor epithelial cells, as well as associated endothelial cells and stroma cells. Large metastases showed central necrosis. Lung metastasis was not observed either by gross or by microscopic examination.

## Discussion

c-Jun has been demonstrated to transduce a mitogenic response and to involve in promoting cell growth as a single gene or in cooperation with an activated ras gene [15,16]. A critical role for c-Jun in the migration and invasion characteristics of human breast cancer cell line in *in vitro *experiment has been demonstrated [25]. The contribution of c-Jun to metastatic phenotype in breast cancer cells has also been studied in *in vitro *studies [25]. However, the role of c-Jun in metastasis of human breast cancer *in vivo *is currently unknown. In the present study, by utilizing MCF-7 cell line as a cell model the effects of c-Jun overexpression were studied in both *in vitro *and *in vivo *experiments to further investigate the direct involvement of c-Jun in tumorigenesis and metastasis.

c-Jun acts as a positive regulator of the cell cycle [30,31]. Our data indicated that ectopic overexpression of c-Jun promoted the growth of MCF-7 cells and led to a significant increase in the percentage of cells in S phase. It is possible that uncontrolled cell proliferation is an important first step in tumorigenesis. Overexpression of c-Jun in transfected MCF-7 cells results in increased motility and invasiveness. Interestingly, introduction of c-Jun gene alone into weakly invasive MCF-7 cells led to the transfected cells capable of metastasizing to the nude mouse liver following tail vein injection. For many years it had been reported that many malignant human tumors did not metastasize in the nude mouse. The production of metastasis mostly depends on the intrinsic tumor cell properties. Our data confirms that overexpression of c-Jun contributes to a more invasive phenotype in MCF-7 cells. This study is the first reported example of c-Jun overexpression inducing liver metastasis of MCF-7 cells in nude mice.

Progression of breast cancer is often accompanied by changes of gene expression pattern and requires an accumulation of metastasis supporting genetic modifications. AP-1 is thought to play a central role in reprogramming of the gene expression pattern and regulating the expression of genes directly necessary for cell invasion [27,32]. Several AP-1-responsive genes with roles in facilitating the invasion of malignant cells have been described [33-35]. The critical target genes activated by c-Jun/AP-1 in metastasis of MCF-7/c-Jun cells remain to be defined.

## Conclusion

In summary, the present study demonstrates an interesting relationship between c-Jun expression and increased property of adhesion, migration and liver metastasis of MCF-7 breast cancer cells. The results provide further evidence that c-Jun alone affects many aspects of the breast cancer phenotype and is involved in the metastasis of breast cancer. This finding also opens an opportunity for development of anti-c-Jun strategies in breast-cancer therapy.

## Competing interests

The author(s) declare that they have no competing interests.

## Authors' contributions

YZ constructed the expression plasmid, carried out immunohistochemical study, performed statistical analysis and participated in writing the manuscript. XP, BF participated in the design of the study and coordinated the study. MS participated in *in vivo *studies. LY, LQ, YS evaluated the immunohistochemical data. GY, HZ, MH participated in the proliferation, adhesion and motility assays. MY performed flow cytometry analysis. NG participated in the design of the study, supervised the laboratory work, and drafted the manuscript. All authors read and approved the final manuscript.

## Pre-publication history

The pre-publication history for this paper can be accessed here:


